# Supplementary material for: Financing sustainability: Applying the BIOFIN framework to government investments in conserving native and indigenous livestock breeds in central India
Source: PLoS One. 2025 Aug 25;20(8):e0330728. doi: 10.1371/journal.pone.0330728 (PMC12377603; doi:10.1371/journal.pone.0330728)
Supplement: S1 Table — (DOCX) [file pone.0330728.s001.docx]

S1 Table. Status of schemes in the animal husbandry department for the conservation of native and indigenous livestock in Madhya Pradesh, India during 2016 to 2022.

| Status of schemes | Conservation | Sustainable | Awareness | Policy | ABS | Total |
| --- | --- | --- | --- | --- | --- | --- |
| Schemes active during 2016-2017 | 15 | 15 | 9 | 0 | 0 | 39 |
| Newly introduced schemes during 2016-2017 | 03 | 25 | 0 | 0 | 0 | 28 |
| Total active schemes during 2016-2017 | 18 | 40 | 9 | 0 | 0 | 67 |
| Discontinued schemes during  2016 to 2022 | 07 | 13 | 04 | 0 | 0 | 24 |
| Total active schemes during 2021-22 | 12 | 27 | 4 | 0 | 0 | 43 |
| Percentage change in schemes from 2016 to 2022 | -20% | +80% | -56% | 0 | 0 | +10.25% |
