# Supplementary material for: Financing sustainability: Applying the BIOFIN framework to government investments in conserving native and indigenous livestock breeds in central India
Source: PLoS One. 2025 Aug 25;20(8):e0330728. doi: 10.1371/journal.pone.0330728 (PMC12377603; doi:10.1371/journal.pone.0330728)
Supplement: S2 Table — (DOCX) [file pone.0330728.s002.docx]

S2 Table. The number of schemes with Biodiversity Attributed to conserving native and indigenous livestock in the animal husbandry department of Madhya Pradesh, India from 2016 to 2022.

| Biodiversity Attribution | Conservation | | Sustainable | | Awareness | | Policy | | ABS | | Total | | | Percentage of  total Schemes | | | Diff in Changes | |
| --- | --- | --- | --- | --- | --- | --- | --- | --- | --- | --- | --- | --- | --- | --- | --- | --- | --- | --- |
|  | 2016-17 | 2021-22 | 2016-17 | 2021-22 | 2016-17 | 2021-22 | 2016-17 | 2021-22 | 2016-17 | 2021-22 | 2016-17 | 2021-22 | 2016-17 | | 2021-22 | From 2016-17 to 2021-22 | |  |
| Direct  100-90  (95%) | 7 | 4 | 1 | 0 | 1 | 0 | 0 | 0 | 0 | 0 | 9 | 4 | 23.07 | | 9.30 | -13.77 | |  |
| Indirect Very High  90-75  (82.5%) | 3 | 4 | 0 | 0 | 0 | 0 | 0 | 0 | 0 | 0 | 3 | 4 | 7.70 | | 9.30 | +1.6 | |  |
| Indirect High  75-50  (62.5%) | 0 | 2 | 0 | 0 | 0 | 0 | 0 | 0 | 0 | 0 | 0 | 2 | 00 | | 4.65 | +4.65 | |  |
| Indirect Medium  50-25  (37.5%) | 3 | 2 | 1 | 4 | 7 | 3 | 0 | 0 | 0 | 0 | 11 | 9 | 28.21 | | 20.93 | -7.28 | |  |
| Indirect Low  25-5  (15%) | 2 | 0 | 7 | 22 | 1 | 1 | 0 | 0 | 0 | 0 | 10 | 23 | 25.64 | | 53.49 | +27.85 | |  |
| Indirect Marginal  5-0  (2.5%) | 0 | 0 | 6 | 1 | 0 | 0 | 0 | 0 | 0 | 0 | 6 | 1 | 15.38 | | 2.33 | -13.05 | |  |
| Total | 15 | 12 | 15 | 27 | 9 | 4 | 0 | 0 | 0 | 0 | 39 | 43 | 100 | | 100 |  | |  |
