# Supplementary material for: Financing sustainability: Applying the BIOFIN framework to government investments in conserving native and indigenous livestock breeds in central India
Source: PLoS One. 2025 Aug 25;20(8):e0330728. doi: 10.1371/journal.pone.0330728 (PMC12377603; doi:10.1371/journal.pone.0330728)
Supplement: Attachment — Submitted filename: Response to reviwers.docx [file pone.0330728.s004.docx]

**Financing Sustainability: Applying the BIOFIN Framework to Government Investments in Conserving Native and Indigenous Livestock Breeds in Central India.**

**Reviewer #1:**

**Comments 1:** Overall, the article is well-written, informative, and communicates the key findings and implications. The manuscript presented in an intelligible fashion, with structured statistical analysis and written in standard English, but needs some language correction and recommendations for publication.

**Our response:** We are grateful to the reviewer for taking his valuable time to look at our work. We thoroughly reviewed the manuscript and made significant language and grammar edits throughout that further improved its readability.

**Reviewer #2:**

**Comments 1:** Line 63-66: mention some countries and need citation.

**Our response:** We have now added a list of the countries and the associated citations in the revised manuscript. Please see lines 62 to 65. Thank you.

**Comments 2:** Line 78: delete the phrase “natural resource”

**Our response:** Suggestion accepted. We have removed the phrase.

**Comments 3:** Line 113-114: what do you mean changes in land use

**Our response:** We have removed the phrase.

**Comments 4:** What are your criteria to say native or indigenous?

**Our response:** We have now added a paragraph (lines 119-121) to explain the criteria we used to classify a species into native or indigenous. Accordingly, both native and indigenous breeds originated in India. However, native breeds are specific to Madhya Pradesh (our study area), while indigenous breeds may also come from other parts of India.

**Comments 5:** Line 119: Capra aegagrus hircus?

**Our response:** We removed the word aegagrus.

**Comments 6:** Line 126: 7,48,92,284.68?

**Our response:** We understand this way of representing large numbers could be confusing to readers. Therefore, we have now written this number as 74.9 million Indian rupees. Line number 129.

**Comments 6:** What is the effect or output from each scheme? e.g Make a comparison in population and productivity trend for conserved breed between initial year and current year.

**Our response:** Although the impact assessment of each scheme is an important question, it was beyond the scope of our study. Our study objectives focused solely on studying the financial investment for native breed conservation using the Biofin Methodology.

**Comments 7:** Which strategy or approach is used for conservation?

**Our response:** The conservation criteria—effects and outcomes—had already been addressed by the Madhya Pradesh Animal Husbandry Department, so our study did not include them.

**Comments 7:** how many skilled personnel were produced from awareness scheme?

**Our response:** The awareness schemes were constituted to aware the farmers and community only to promote the rearing of native and indigenous livestock. The awareness programme does not cover the skill development.

**Comments 8:** What type of breeding strategy implemented or implementing?

**Our response:** We have incorporated this information in line numbers 350 to 360. The Policy and Institutional Review highlights various strategies and approaches for conservation, including Livestock Breeding Farms, Gau Samvardhan, Livestock Sanctuaries, the National Livestock Mission, the Intensive Livestock Development Programme, and the induction of both small and large animals. Additionally, Vidyasagar Gau Samvardhan, Poultry Farms for breed conservation, Sheep Farms for sheep conservation, and the Rashtriya Krishi Vikas Yojna for nucleus herd development and the promotion of indigenous or native breed sorted semen were purposefully adopted to address conservation challenges for making strategies.

**Comments 9:** If there is incentive for participant farmers; what type of incentive,

**Our response:** There are no such direct incentives for farmers. However, continuous awareness and consecutive training may be considered as incentive.

**Comments 10:** What are the criteria for budget allocation for each scheme?

**Our response:** The criterion of budget allocation depended on the requirement of the fund proposed by the department as per the requirement to fulfil the objectives of programmes and schemes.

**Comments 11:** Explain in detail for the sustainable issues (discontinued projects such as: calf rearing for cows and buffaloes, the National Kamdhenu Breeding Centre (NKBC), Embryo Transfer Technology (ETT), and the Animal Breeding Centre were discontinued. In the sustainable category, 27 schemes were evaluated to address sustainable issues, but key schemes like National Animal Health & Disease Prevention and Fodder Production were discontinued.) and others.

**Our response:** We have incorporated this information in line number 365 to 371. The sustainable issues for the discontinued schemes, the 25 New schemes were new launched according to the policy of the government to fulfil the gaps of the discontinued schemes. Among them the schemes like Division and District level AH and Dairy, Strengthening of VET Hospitals and dispensaries, Est. Veterinary Council of India, Mob VET Unit Central, Est of New Vet Hospital and Dispensary Strengthening of VET Hospitals Mob VET Services, Vet Hosp and Other Infra Development, etc have been newly launched.
