# Supplementary material for: Financing sustainability: Applying the BIOFIN framework to government investments in conserving native and indigenous livestock breeds in central India
Source: PLoS One. 2025 Aug 25;20(8):e0330728. doi: 10.1371/journal.pone.0330728 (PMC12377603; doi:10.1371/journal.pone.0330728)
Supplement: Attachment — Submitted filename: Response_to_reviwers_auresp_2.docx [file pone.0330728.s005.docx]

**Financing Sustainability: Applying the BIOFIN Framework to Government Investments in Conserving Native and Indigenous Livestock Breeds in Central India.**

**Reviewer #1:**

**Comments 1:** Line 123-129: Need appropriate citation for listed native and indigenous breeds.

**Our response:** We are grateful to the reviewer for taking his valuable time to look at our work. We have now added the associated citations in the revised manuscript. Please see lines 123 to 129. Thank you

**Comments 2:** From a total of identified 67 schemes from 2016 to 2022, how many of them are successful? How many discontinued? For which breed of conservation is more successful?

**Our response:** Since 2016, a total of 43 schemes have continued successfully, while 24 were discontinued. Please see lines 252 to 255. These schemes have worked well for the conservation of native and indigenous livestock breeds. Some of the successful breeds include Murrah buffalo, Gir, Sahiwal, Rathi, Red Sindhi cattle, Ongole cattle, Punganur cattle, Malnad Gidda cattle, Deoni cattle, Hallikar cattle, and Kadaknath for poultry conservation. We have included in line number 356 to 359.

**Comments 3:** If conservation criteria, effects and outcomes had already been addressed by the Madhya Pradesh Animal Husbandry Department, include the information in the background (introduction) section.

**Our response:** Suggestion accepted. We have included in the background. We have included in line number 133 to 137. The Animal Husbandry Department has assessed the impacts and outcomes of its various initiatives, with a particular focus on disease control, infrastructure development, and the enhancement of animal welfare and productivity. Additionally, the department has advanced breeding programmes and genetic improvement initiatives, complemented by targeted training and awareness campaigns.

**Comments 4:** To get good information about the conservation program and to understand BIOFIN for your target population: you should include information such as number of participant farmers or experts, number of animals for each breed, number of farms……etc in table form in the methodology section

**Our response:** The objectives of the study are to find out the trend of financing for implemented schemes by department of animal husbandry for using BIOFIN methodology conservation of the indigenous/ native breeds of livestock. The issue does not relate with the objectives of the study therefore the no of farmers or experts not included. As per suggestion In Methodology section livestock population included. The target population for the schemes implemented by the Department of Animal Husbandry Madhya Pradesh is the 1.87 crore (18.8 million) (1.70 million indigenous population) cattle population and 1.03 crore the Buffalo population (19 Animal Census). We have included in line number 189 to 191.

**Comments 5:** Does the breeding strategy is natural or artificial? using sorted semen for which breed? What is the distribution modality of native and indigenous breeds??

**Our response:** As per the Policy and institutional review of the schemes, both the natural and artificial insemination technology were used for successful breeding purposes. It includes the semen of specific native and indigenous breeds of cattle and buffalo were used in the respective breeds in their native tracts in the state (i.e. Tharparkar in Rajasthan, Gir in Gujarat). We have included in line number 171 to 174.

**Comments 6:** What is the reason for discontinued of some conservation schemes

**Our response:** The reasons of discontinued conservation schemes are (i) achievements of the scheme's objectives and targets, (ii) financial constraints (discontinued due to budget limitations or reallocation of funds) to other priorities in the livestock sector for the other conservation schemes), (iii) shifting priorities as per the state policy according to relevance. We have included in line number 387 to 390.

**Comments 7:** Use consistence reference format

**Our response:** As per suggestion references format corrected.
